# Supplementary material for: Dynamics-informed priors (DIP) for neural mass modelling
Source: Imaging Neurosci (Camb). 2026 May 29;4:IMAG.a.1250. doi: 10.1162/IMAG.a.1250 (PMC13224311; doi:10.1162/IMAG.a.1250)
Supplement: Supplementary Material [file IMAG.a.1250_supp.pdf]

Supplementary Material

1 Supplementary 1

1.1 Neural mass equations

The model used in **Study 1** and **Study 2** was from the SPM12 toolbox and is described in detail in Moran et al., 2007. It comprises three neuronal populations considered as single entities, as illustrated in the schematic in **Figure S1**. The spiny stellate cell population receives external inputs, I, and excitatory inputs from pyramidal cells that are scaled by the connectivity parameter G_1. Their dynamics are formalised as follows:

$$\frac{dx_1}{dt} = x_4$$

$$\frac{dx_4}{dt} = \frac{H_e\left(\left(A_1 + A_3\right)S(x_9) + G_1 S(x_9) + I\right) - 2x_4 - \frac{x_1}{T_e}}{T_e}$$

where x_1 is the average post-synaptic potential (PSP) and x_4 the output signal of the population. $S(x)$ is the sigmoid transformation of the membrane potential into a firing rate, described as follows:

$$S(x) = \frac{1}{\left(1 + e^{-R_1(x-R_2)}\right)} - \frac{1}{\left(1 + e^{R_1 R_2}\right)}$$

where R_1 and R_2 capture the slope and offset of the sigmoid function, respectively.

The excitatory pyramidal cell population receives excitatory afferents from spiny stellate cells scaled by the connectivity parameter G_2, and inhibitory inputs scaled by the connectivity parameter G_4:

$$\frac{dx_2}{dt} = x_5$$

$$\frac{dx_5}{dt} = \frac{H_e\left(\left(A_2 + A_3\right)S(x_9) + G_2 S(x_1)\right) - 2x_5 - \frac{x_2}{T_e}}{T_e}$$

$$\frac{dx_3}{dt} = x_6$$

$$\frac{dx_6}{dt} = \frac{H_i G_4 S(x_{12}) - 2x_6 - \frac{x_3}{T_i}}{T_i}$$

$$\frac{dx_9}{dt} = x_5 - x_6$$

where x_2, x_3 are the PSPs, x_5, x_6 the firing rates, and x_9 the depolarisation depending on excitatory and inhibitory PSPs (EPSPs and IPSPs).

The inhibitory subpopulation receives afferent input from the excitatory pyramidal cell population scaled by the connectivity parameter G_3. The inhibitory subpopulation also receives recurrent inhibitory inputs scaled by the connectivity parameter G_5. The associated state equations are as follows:

$$\frac{dx_7}{dt} = x_8$$

$$\frac{dx_8}{dt} = \frac{H_e\left(\left(A_1 + A_3\right)S(x_9) + G_3 S(x_9)\right) - 2x_8 - \frac{x_7}{T_e}}{T_e}$$

$$\frac{dx_{10}}{dt} = x_{11}$$

$$\frac{dx_{11}}{dt} = \frac{H_i G_5 S(x_{12}) - 2x_{11} - \frac{x_{10}}{T_i}}{T_i}$$

$$\frac{dx_{12}}{dt} = x_8 - x_{11}$$

where x_7, x_{10} are the PSPs, x_8, x_{11} the firing rates, x_{12} the depolarisation depending on EPSPs and IPSPs.

Finally, x_{13} plays a role attributable to a slow potassium conductance for the modulation of spike generation (Moran et al., 2007, 2013), as follows:

$$\frac{dx_{13}}{dt} = \frac{4S(x_1) - x_{13}}{T_k}.$$

The hidden neural state variables

$$\mathbf{x} = \begin{pmatrix} x_1 \\ x_2 \\ \vdots \\ x_{13} \end{pmatrix}$$

denoted by the vector $\mathbf{x} \in \mathbb{R}^{13}$, are mapped to the measured EEG data via the observation function:

$$g(\mathbf{x}) = f(\mathbf{x}, u, \boldsymbol{\theta}) + Cu.$$

where f is the nonlinear neuronal model describing the evolution of the state variables over time, $\boldsymbol{\theta}$ is the set of parameters influencing how the states evolve, g is the function that maps hidden neural states x to the observations. C is a vector describing the system's modulation by extrinsic inputs, and u is the scalar input. Model parameters are reported in **Table S1**.

Table S1: **Parameter Priors and Bounds**. Prior parameter distributions were the default SPM12 priors, μ is the mean and σ is the variance. The bounds on the parameters explored via the genetic algorithm (GA) are specified and reported here as [lower bound; upper bound]. Parameters are log-scaled to ensure positivity (Friston et al., 2019). Parameters T_k and H_i were fixed by default in the SPM model (see **Supplementary 1.4**).

Parameters	DCM priors $P(\boldsymbol{\theta})$	GA bounds
R_1 static nonlinearity, slope	$\mu = 1, \sigma = 0.1250$	$[0.2431; 4.1132]$
R_2 static nonlinearity, offset	$\mu = 2, \sigma = 0.1250$	$[0.4862; 8.2264]$
T_e excitatory synaptic time constant	$\mu = 0.004, \sigma = 0.1250$	$[0.000972; 0.0165]$
T_i inhibitory synaptic time constant	$\mu = 0.016, \sigma = 0.1250$	$[0.0039; 0.0658]$
T_k potassium synaptic time constant	$\mu = 0.5120, \sigma = 0$	0.5120
G_1 pyramidal to stellate population connectivity	$\mu = 128, \sigma = 0.0625$	$[46.8725; 347,9401]$
G_2 stellate to pyramidal population connectivity	$\mu = 128, \sigma = 0.0625$	$[34.1660; 347,9401]$
G_3 pyramidal to inhibitory population connectivity	$\mu = 64, \sigma = 0.0625$	$[23.4362; 183.0543]$
G_4 inhibitory to pyramidal population connectivity	$\mu = 64, \sigma = 0.0625$	$[23.4362; 174.0745]$
G_5 inhibitory self-connectivity	$\mu = 4, \sigma = 0.0625$	$[1.3521; 12.0371]$
H_e excitatory synaptic gain	$\mu = 8, \sigma = 0.0625$	$[2.6449; 21.7463]$
H_i inhibitory synaptic gain	$\mu = 32, \sigma = 0$	32
A_1 extrinsic forward connectivity	$\mu = 32, \sigma = 0.5$	$[1.2367; 541.3878]$
A_2 extrinsic backward connectivity	$\mu = 16, \sigma = 0.5$	$[0.9456; 312.2764]$
A_3 extrinsic lateral connectivity	$\mu = 4, \sigma = 0.5$	$[0.1510; 67.6735]$
D_e extrinsic propagation delay	$\mu = 0.002, \sigma = 0.0625$	$[0.000736; 0.0054]$
D_i intrinsic propagation delay	$\mu = 0.016, \sigma = 0.0312$	$[0.0079; 0.0348]$

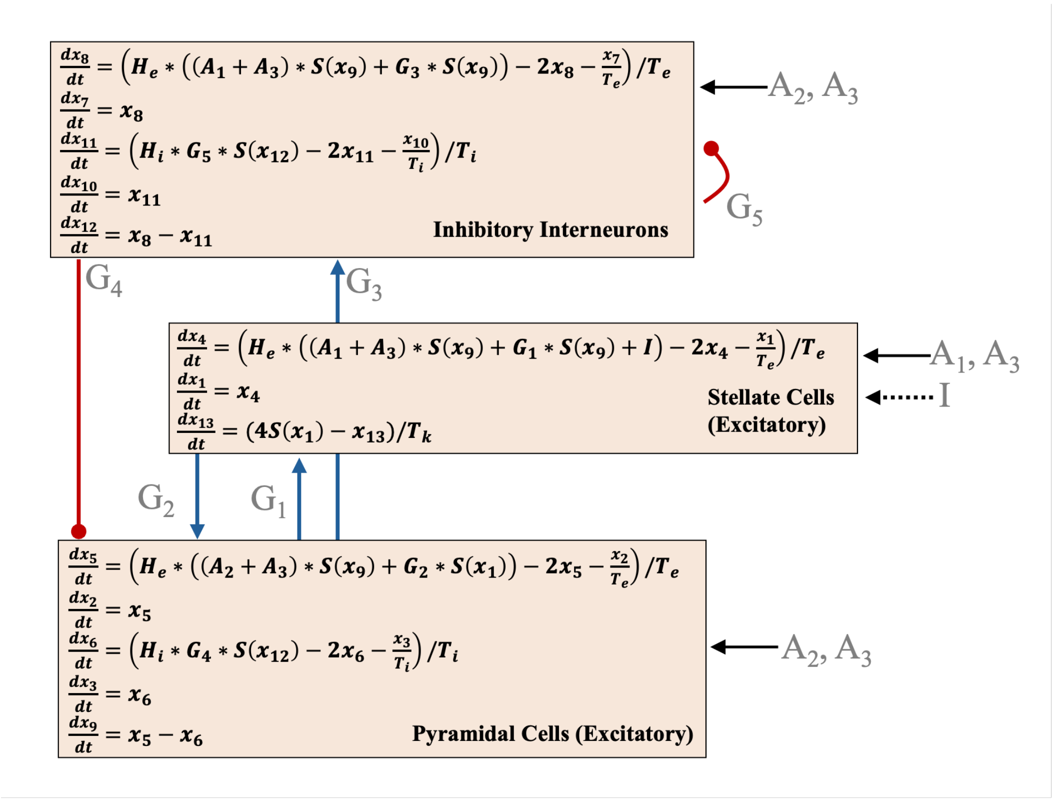

Figure S1: **Schematic diagram of the model**. The model comprises three interconnected neural populations, whose activity is described my average membrane potential and firing rate variables.

1.2 Spectral response

Local linearity assumptions were adopted to circumvent the challenges of parameter estimation typical of nonlinear models and to enhance computational efficiency. The sigmoid function $S(x)$ is approximated via Taylor expansion around the steady state $\mathbf{x_0}$ using the default SPM12 `spm_dcm_neural_x` function. At $x = 0$, the firing rate is described by:

$$S(\mathbf{x_0}) = \frac{1}{(1 + e^{-R_1(\mathbf{x_0} - R_2)})} - \frac{1}{(1 + e^{R_1 R_2})},$$

meaning that all state variables have resting values of zero. The dynamics of the model around this point are dependent on the slope of the sigmoid function at $\mathbf{x_0}$,

$$\frac{dS}{dx} = \frac{R_1 e^{R_1 R_2}}{(1 + e^{R_1 R_2})^2}.$$

The linear approximation of the system is:

$$\dot{x} = A\mathbf{x}(t) + Cu, \quad y_{obs} = G\mathbf{x}(t)$$

where

$$A_{i,j} = \left.\frac{\partial \mathbf{f_i}}{\partial \mathbf{x_j}}\right|_{\mathbf{x}=\mathbf{x_0}}$$

is the Jacobian matrix representing the linear behaviour of the system around the steady state, and G is a row vector mapping the hidden states to the observables, quantifying the contribution of the states $\mathbf{x}$ to the observed output y_{obs}. This mapping is described as follows:

$$G \in \mathbb{R}^{13}, \quad G_i = \begin{cases} 1, & \text{if } i = 9 \\ 0, & \text{if } i \neq 9 \end{cases}.$$

The Laplace transform was applied to the linear equations (Moran et al., 2007; Oppenheim et al., 1983) utilising the SPM12 `spm_csd_mtf` function to generate the power spectral density (PSD) of the model as follows:

$$Y(s) = G(sI - A)^{-1}C$$

where s represents the complex Laplace variable, A is the Jacobian matrix evaluated at $\mathbf{x_0}$, G is the vector mapping the hidden states to the observed signal, C is the input mapping, indicating how the inputs affect the system, and I is the identity matrix.

This PSD is modulated by different sources of noise (endogenous neuronal fluctuations, measurement noise and data filtering that contributed to producing the observed PSD). This is conveyed by explicit parameterisation (Friston et al., 2015; Moran et al., 2009; Novelli et al., 2024; Razi et al., 2015). For details on the structure of this noise term and the likelihood function, please refer to the section below (**Supplementary 1.3**). Ultimately, the spectral response is log-transformed and becomes the predicted observation updated within the variational inversion and the GA optimisation. The respective SPM12 functions were adapted to account for this log-transformation. Throughout this work, model and data PSD refer to the log-transformed versions used for all inversion schemes.

1.3 Observation noise

The observation function that generates the predicted spectral responses has two main contributions. One is neuronal, i.e., the pyramidal cell depolarisation, and the other is observation noise (**Supplementary 1.2**). Noise is modelled through parameterisation (**Table S2**; Friston et al., 2015; Razi et al., 2015; `spm_csd_mtf_gu` function from SPM12) of the observation function, modulating the predicted spectral response as follows:

$$Y_u(\omega) = e^{a_1} \cdot \omega^{-e^{a_2}} \cdot e^{Y_d d}$$

$$Y_n(\omega) = e^{b_1} \cdot \omega^{-e^{b_2}}$$

$$Y_s(\omega) = e^{c_1} \cdot \omega^{-e^{c_2}}$$

$$Y_2 = \hat{y_L} \cdot \rho(Y_u)$$

$$Y_{\text{norm}} = \frac{Y_2}{\int Y_2 \, df}$$

$$Y_{\log} = \log(Y_{\text{norm}})$$

$$\hat{y_m} = Y_{\log} + Y_s + Y_n$$

where $\hat{y_L}$ is the PSD of the model obtained via the Laplace transform. The parameters $a_1, a_2, b_1, b_2, c_1, c_2, d$ serve as modulations of the the predicted PSD. Specifically, a_1 is the amplitude of neuronal fluctuations, or innovations, i.e., spontaneous noisy activity, a_2 is the exponent of these innovations, b_1, b_2, c_1, c_2 capture amplitude and exponent of non-specific and specific channel noise (noise from nearby sources or from the recording channel). Parameters d (i.e.,d_1, d_2, d_3, d_4) represent additional neuronal noise. Thus, y_u, y_n and y_s are spectral modulation vectors generated from noise parameters, considering that ω represents a vector of frequency bins dependent on the PSD of data and that Y_d parameterises variations in individual spectral bins. Y_2 is an intermediate PSD adjusted to account for noise, and obtained by multiplying the PSD $\hat{y_L}$ by the diagonal of the noise parameterisation matrix Y_u, where ρ is the diagonal function. The

PSD is also modulated by v_ω, representing effects of filtering, which is formalised as follows:

$$v_\omega = e^{f_1 + \frac{f_2 \omega}{n}}, \quad \text{for } \omega = 1, 2, \ldots, n$$

$$h = \hat{y_m} \odot v_\omega,$$

f_1, f_2 are the parameters for data filtering, f is the frequency-dependent filter, n is the total number of frequency bins, $\odot$ denotes element-wise multiplication, and h is the log-transformed model PSD, representing the observed (or measured) output recapitulating empirical data. Therefore, the predicted observation, dependent on the parameters $\boldsymbol{\theta}$ and entering the inversion scheme, is:

$$\hat{y} = h(\boldsymbol{\theta}) + \epsilon,$$

with ϵ being the noise.

This work treats real and predicted spectra in log space. The spectral likelihood, representing the probability of observing the PSD $y(\omega)$ for parameters $\boldsymbol{\theta}$ is defined from (Friston et al., 2003) as:

$$\ln P(\log(y) \mid \boldsymbol{\theta}) = -\frac{1}{2}(\log(y) - \hat{y})^T C_\epsilon^{-1} (\log(y) - \hat{y})$$

where $\log(y)$ is the log PSD of the data at frequency ω, $\hat{y}$ is the log PSD of the model at ω, given the vector of parameters $\boldsymbol{\theta}$ and C^{-1} is the noise covariance).

Table S2: **Priors and bounds on noise parameters**. Prior parameter distributions were the default SPM12 priors for the model, μ is the mean and σ is the variance of the normal distributions. The bounds on the parameters explored via the genetic algorithm (GA) are reported as [lower bound; upper bound]. Parameters were log-scaled to ensure positivity.

Parameters	DCM priors	GA bounds
a_1 neuronal fluctuations, amplitude	$\mu = 1, \sigma = 0.0078$	$[0.7022; 1.4242]$
a_2 neuronal fluctuations, exponent	$\mu = 1, \sigma = 0.0078$	$[0.5571; 1.4242]$
b_1 channel noise (non-specific), amplitude	$\mu = 1, \sigma = 0.0078$	$[0.6856; 1.4242]$
b_2 channel noise (non-specific), exponent	$\mu = 1, \sigma = 0.0078$	$[0.7022; 1.4279]$
c_1 channel noise (specific), amplitude	$\mu = 1, \sigma = 0.0078$	$[0.6856; 1.4242]$
c_2 channel noise (specific), exponent	$\mu = 1, \sigma = 0.0078$	$[0.7022; 1.4279]$
d_1, d_2, d_3, d_4 neuronal fluctuations	$\mu = 1, \sigma = 0.0078$	$[0.6953; 1.4242]$
		$[0.7022; 1.4495]$
		$[0.7022; 1.5543]$
		$[0.6702; 1.4242]$
f_1, f_2 data filtering	$\mu = 1, \sigma = 0.0156$	$[0.6065; 1.6487]$
		$[0.5156; 1.6487]$

1.4 Full and reduced models

The 'out-of-the-box' implementation of the SPM12 LFP model does not treat the parameters Tk and Hi as free parameters (**Table S1**). The present work maintained this formulation, as it sought to compare the performance of the standard DCM to that of DCM with dynamics-informed priors (DIP-DCM) on an unmodified SPM model. This 'reduced' model, where Tk and Hi are fixed, was compared to the 'full' model, which treats all neuronal parameters as free parameters (**Table S3**, **Figure S2**).

Full and reduced models were compared via Bayesian model comparison. Considering that models at within each group had equal probability, $1/m$ with $m = 1, ..., 500$, the group-level log model evidence was calculated as follows:

$$log \sum_{m=1}^{500} e^{F_m},$$

where F_m is variational free energy of each mth model in the group. Thus, the log Bayes factor ($\log BF$) for Bayesian model comparison was:

$$\log BF = \log \sum_{m=1}^{500} e^{F_{reduced,m}} - \log \sum_{m=1}^{500} e^{F_{full,m}}.$$

$\log BF > 3$ corresponds to statistical significance at $\alpha = 0.05$ (since $e^3 = 20$, a difference of 3 in log evidence corresponds to a ratio of $20^{-1} = 0.05$). Therefore, the full and reduced models had the same

evidence, as $\log BF \approx 0$ for all experimental conditions (**Table S4**). However, since the full model required substantially longer computation times to generate the same dynamics (see **Figure 3B** in the main paper), this paper adopted the default reduced model.

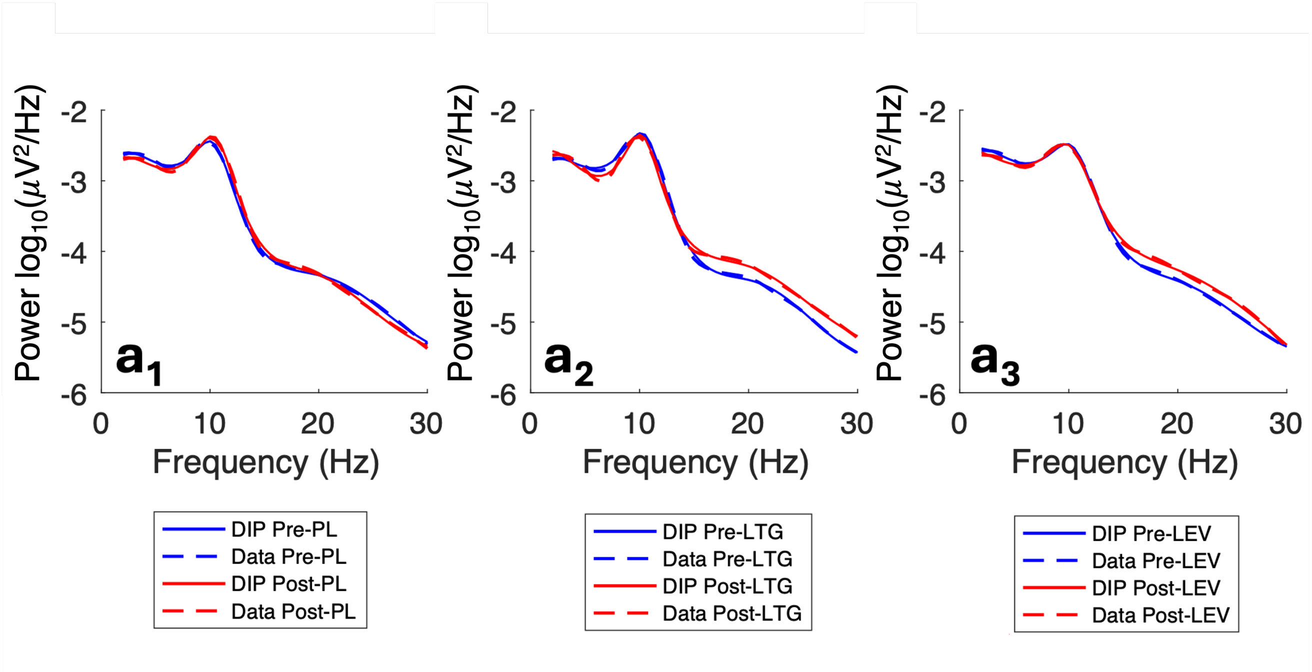

Figure S2: **Full LFP model**. Spectra of data and model for each experimental group: placebo (PL, a_1), lamotrigine (LTG, a_2) and levetiracetam (LEV, a_3). Data was from Biondi et al., 2022. *Abbreviations*: DIP, dynamics-informed priors.

Table S3: **Parameter priors and bounds for the full LFP model**. Parameter priors were the default SPM12 priors, μ is the mean and σ is the variance. Parameter bounds utilised for the genetic algorithm (GA) are reported as [lower bound; upper bound] and are biologically realistic.

Parameters	DCM priors	GA bounds
R_1 static nonlinearity, slope	$\mu = 1, \sigma = 0.1250$	$[0.2431; 4.1132]$
R_2 static nonlinearity, offset	$\mu = 2, \sigma = 0.1250$	$[0.4862; 8.2264]$
T_e excitatory synaptic time constant	$\mu = 0.004, \sigma = 0.1250$	$[0.000972; 0.0165]$
T_i inhibitory synaptic time constant	$\mu = 0.016, \sigma = 0.1250$	$[0.0039; 0.0658]$
T_k potassium synaptic time constant	$\mu = 0.5120, \sigma = 0.125$	$[0.1017, 2.1060]$
G_1 pyramidal to stellate population connectivity	$\mu = 128, \sigma = 0.0625$	$[46.8725; 347.9401]$
G_2 stellate to pyramidal population connectivity	$\mu = 128, \sigma = 0.0625$	$[34.1660; 347.9401]$
G_3 pyramidal to inhibitory population connectivity	$\mu = 64, \sigma = 0.0625$	$[23.4362; 183.0543]$
G_4 inhibitory to pyramidal population connectivity	$\mu = 64, \sigma = 0.0625$	$[23.4362; 174.0745]$
G_5 inhibitory self-connectivity	$\mu = 4, \sigma = 0.0625$	$[1.3521; 12.0371]$
H_e excitatory synaptic gain	$\mu = 8, \sigma = 0.0625$	$[2.6449; 21.7463]$
H_i inhibitory synaptic gain	$\mu = 32, \sigma = 0.0625$	$[11.7721; 91.7196]$
A_1 extrinsic forward connectivity	$\mu = 32, \sigma = 0.5$	$[1.2367; 541.3878]$
A_2 extrinsic backward connectivity	$\mu = 16, \sigma = 0.5$	$[0.9456; 312.2764]$
A_3 extrinsic lateral connectivity	$\mu = 4, \sigma = 0.5$	$[0.1510; 67.6735]$
D_e extrinsic propagation delay	$\mu = 0.002, \sigma = 0.0625$	$[0.000736; 0.0054]$
D_i intrinsic propagation delay	$\mu = 0.016, \sigma = 0.0312$	$[0.0079; 0.0348]$

Table S4: **Bayesian model comparison between the reduced and full model**. For each of the six sub-datasets (pre- and post-PL, LTG, LEV) from Biondi et al., 2022, the reduced model had variational free energy $F_{reduced}$, and the full model had variational free energy F_{full}. Each model was a group of m models, as defined by the respective dynamics-informed priors. *Abbreviations:* PL, placebo; LTG, lamotrigine; LEV, levetiracetam.

Dataset	$F_{reduced} - F_{full}$
$Pre - PL$	0.1286
$Post - PL$	-0.0442
$Pre - LTG$	-0.1294
$Post - LTG$	-0.5011
$Pre - LEV$	0.1926
$Post - LEV$	0.3210

2 Supplementary 2

2.1 Dynamic causal modelling

Prior beliefs on parameter values (**Table S1**) entered the traditional VL routine to estimate an updated posterior parameter density and a free energy approximation to model evidence computed based on the estimated parameters (see Friston et al., 2003; Parr et al. T. and Friston, 2022; Zeidman et al., 2023 for in-depth derivation). By Bayes' product rule, the true posterior is:

$$P(\boldsymbol{\theta}|y) = \frac{P(y|\boldsymbol{\theta})P(\boldsymbol{\theta})}{P(y)},$$

where y is the PSD of the data used to infer the parameters $\boldsymbol{\theta}$, $P(\boldsymbol{\theta})$ are the prior beliefs, $P(y|\boldsymbol{\theta})$ is the likelihood, and $P(y)$ is model evidence, or marginal likelihood. Considering that the model evidence $P(y)$ cannot be calculated, the true posterior distribution $P(\boldsymbol{\theta}|y)$ is approximated with a simpler distribution $Q(\boldsymbol{\theta})$, which, under the Laplace assumption, is considered to be Gaussian and specified as expectation (mean) and variance. The marginal likelihood, or model evidence, is approximated by free energy, defined as follows:

$$F[Q, y] = D_{KL}[Q(\boldsymbol{\theta})||P(\boldsymbol{\theta})] - E_Q(\boldsymbol{\theta})[\ln P(y|\boldsymbol{\theta})],$$
$$F[Q, y] \approx P(\boldsymbol{\theta}),$$

where free energy $F[Q, y]$ is a functional of the approximate posterior, Q, and of the data y. The first term represents model complexity and it is quantified as Kullback–Leibler (KL) divergence (D_{KL}) between the approximate posterior $Q(\boldsymbol{\theta})$ and the prior $P(\boldsymbol{\theta})$. The second term is accuracy and it is the expected value with respect to the variational distribution $Q(\boldsymbol{\theta})$, ($E_Q(\boldsymbol{\theta})$), of the log likelihood ($\log P(y|\boldsymbol{\theta})$).

Thus, parameter estimation is treated as an optimisation problem, where parameters are optimised in the direction of maximum increase of F.

2.2 Genetic algorithm

The genetic algorithm (GA) was adapted from Dunstan et al., 2023 for SPM and DCM compatibility and to suit the amis of this study. Specifically, while in Dunstan et al., 2023 the approach is nonlinear and simulates an EEG time series, the present study simulates a power spectrum using a transfer function

from SPM12, which ensures faster computation. Moreover, the GA hyperparameters were increased from 50 to at least 150 to improve the parameter estimation (as explained in **Supplementary 4**). The population size was increased from 500 to 650, which improved convergence, and the number of replicates was increased from 100 to at least 500, for similar robustness considerations (further details are in **Supplementary 4**). The objective functions were the RMSE between frequency ranges adapted to the data of the present study (see below), while Dunstan et al., 2023 utilised a different nonlinear objective. Finally, the implementation was modified to accommodate for the use of SPM models and SPM functions and to ensure integration with the variational Laplace for DCM.

The GA utilised the NSGA-II variant from the MATLAB `gamultiobj` function (Deb, 2001). The algorithm initialises a population of parameter vectors V, representing solutions to the optimisation problem, defined as:

$$V(t) = \{\boldsymbol{\theta_1}, \boldsymbol{\theta_2}, \ldots, \boldsymbol{\theta_p}\},$$

where p is a population of parameter sets, and $p = 650$. The population V evolves for t generations, with $t = (0, 1, ..., 500)$ for **Study 1** and $t = (0, 1, ..., 150)$ for **Study 2**. Each parameter set is a vector $\boldsymbol{\theta}$ of 27 parameter values θ_j. The initial population is randomly sampled via Latin hypercube as described in (Dunstan et al., 2023), within specified bounds $[lb_j, ub_j]$ (**Table S1**).

The algorithm utilised two objective functions, and thus it can be referred to as a multi-objective GA. The objectives J_1 and J_2 were evaluated on each parameter set in the population $V(t)$ at each generation t, as follows:

$$J_1 = \sqrt{\sum_{\omega \in \Omega_1} (\log(y(\omega)) - \hat{y}(\omega))^2},$$

$$J_2 = \sqrt{\sum_{\omega \in \Omega_2} (\log(y(\omega)) - \hat{y}(\omega))^2}$$

where $\hat{y}$ is the PSD of the model, y is the PSD of the data at the frequencies Ω_1, Ω_2 ranging between 6.5-12.5 Hz, and 11.5-30 Hz, respectively for the Biondi et al., 2022 dataset. These objectives ensure that the alpha and beta frequencies are well-represented. A similar logic was applied to the Shaw et al., 2020 dataset, with Ω_1, ranging between $[10.7 - 65.8]$ Hz and Ω_2 ranging between $[35.6 - 85]$ Hz.

Thus, Parameter sets are selected from the population based on their fitness scores, determined by the

values of each objective. The built-in `selection tournament` selection function randomly chooses groups of four individuals from the current population. The individual with the best fitness score in the group is chosen as a "parent" to produce offspring for the next generation. Offspring is produced via crossover and mutation (`crossover scattered` and `mutation adapt feasible` functions from the matlab toolbox), evolving the population towards increasingly better solutions. Solution rank is measured by Pareto dominance:

$$\boldsymbol{\theta_A} \in P(t_{end}) \text{ Pareto dominates } \boldsymbol{\theta_B} \in P(t_{end}) \text{ if}$$
$$\forall k = 1, 2, f_k(\boldsymbol{\theta_A}) \leq f_k(\boldsymbol{\theta_B}) \text{ and } \exists k = 1, 2 : f_k(\boldsymbol{\theta_A}) < f_k(\boldsymbol{\theta_B}),$$

with $P(t_{end})$ the set of all solutions. This generated a Pareto front of solutions, i.e., a set of points in the 2D objective space where no solution can be improved without worsening the other. From the Pareto front one solution is selected — the solution with smallest Euclidean distance between the two objectives in the objective space. The GA is repeated n times, with $n = 1000$, yielding a set of solutions:

$$S_n := \{\boldsymbol{\theta_1}, \boldsymbol{\theta_2}, \ldots, \boldsymbol{\theta_n}\}.$$

3 Supplementary 3 - Comparisons against alternative initialisation strategies

DIP-DCM was compared to alternative or intermediate initalisation strategies to demonstrate the importance of each methodological choice in the DIP-DCM pipeline, which tries to address a number of different problems. These include problems with the specification of parameter priors, which DIP-DCM resolves by utilising estimates from a GA, problems with the variational approximation and with the local minima that may be encountered during gradient descent, which DIP-DCM addresses by conducting multiple inversions and creating an averaged posterior parameter distribution.

3.1 DCM-VL inversion with different priors.

The effects of increasing the prior variance was studied using a dataset from **Study 1** (**Methods 2.1**). Data was recorded before and after administration of levetiracetam (LEV), showing an increase in spectral power in the beta range (13-30 Hz). The model was inverted via DCM according to the following settings: 1) "standard" means and unit variance; 2) priors derived from the DIP-DCM bounds, where the prior mean is in the middle of the interval and the variance spans 99% of the interval. Spectral fits, goodness

of model fit and posterior inferences are shown in **Figure S3**a_1,a_2, **Table S5** and **Figure S4**.

As observed for the DCM with "standard" priors, the mechanistic inferences derived from the posterior distributions suggest that the spectral effects of LEV cannot be explained by any neural parameter. However, LEV is well known to induce a specific mechanistic modulation of excitatory synaptic activity, impacting the timescale of synaptic response. Therefore, as indicated in **Discussion 4.1-2**, absence of an effect is implausible, demonstrating the importance of using DIP-DCM to detect effects with higher sensitivity.

3.2 A multi-start adaptation of the DCM-VL inversion, which selects only the "best" estimate

The DIP-DCM model inversion pipeline was adapted by selecting the "best" estimate to form a unimodal posterior, with priors fixed and starting points of VL being the GA estimates. The prior variance was either the "standard" variance or a wider variance (**Figure S3**b_1,b_2).

This multi-start adaptation of DIP-DCM did not improve spectral fits nor the inferences on the posterior parameters compared to the default DCM-VL (**Figure S3**b_1,b_2, **Table S5** and **Figure S4**). However, when the priors were widened by setting a variance equal to the one used in DIP-DCM, the goodness of model fits was improved but no change was detected in the posterior distributions.

3.3 A multi-start adaptation of the DCM-VL inversion, which averages posterior estimates by their model-evidence to obtain a multi-modal posterior

The DIP-DCM model inversion pipeline was adapted as above, by fixing the prior and using the GA estimates as starting points for VL, but the posterior distribution was multi-modal and weighted by model evidence. Weights were obtainted from the exponentiated free energy, and normalised such that the sum of all weights was equal to 1 (**Figure S3**c_1,c_2, **Table S5** and **Figure S4**). First, results show that all model evidence-derived weights were equal to $1/n$, with n being the number of priors, proving that all posteriors equally contribute to the final posterior. This also demonstrates that the original DIP-DCM pipeline, which assumes by default that weights are $1/500$, when $n = 500$, was appropriate. Second, results show that utilising the GA estimates as starting points with priors fixed introduces spurious effects that are implausible considering the known effects of LEV, while DIP-DCM gives a more parsimonious

explanation for the effects of LEV in line with the literature.

3.4 DIP-DCM, which uses the variances of the "standard" priors, rather than unit variances

The DIP-DCM model inversion pipeline was adapted by setting the diagonal elements of the covariance matrix to the variances of the "standard" priors, rather than using larger (unit) variances. This setting led to implausible mechanistic inferences for all experimental conditions from **Study 1** (**Figure S5**).

Taken together, results suggest that the GA estimates are best used as priors in the DIP-DCM approach, rather than as starting points. They also show the importance of initiating the inversion from multiple priors, with sufficiently large variances, and the importance of averaging the priors into a multi-modal posterior, in order to escape local minima and identify effects in other plausible regions of parameter space.

Table S5: **Comparisons against alternative initialisation strategies: goddness of model fit**. Root mean square error (RMSE) was calculated between the model and data as a measure of goodness of model fit. Data is reported for both Pre-Levetiracetam (LEV), or Post-LEV conditions.

DCM	DCM, wider	DCM, different prior
$RMSE_{Pre-LEV} = 0.0467$	$RMSE_{Pre-LEV} = 0.0248$	$RMSE_{Pre-LEV} = 0.0248$
$RMSE_{Post-LEV} = 0.0544$	$RMSE_{Post-LEV} = 0.0243$	$RMSE_{Post-LEV} = 0.0364$
GA	DCM-VL, 'best'	DCM-VL, 'best' wider
$RMSE_{Pre-LEV} = 0.0953$	$RMSE_{Pre-LEV} = 0.0467$	$RMSE_{Pre-LEV} = 0.0248$
$RMSE_{Post-LEV} = 0.1006$	$RMSE_{Post-LEV} = 0.0544$	$RMSE_{Post-LEV} = 0.0243$
DIP-DCM	DIP-VL, weighted average	DIP-VL, unweighted average
$RMSE_{Pre-LEV} = 0.0237$	$RMSE_{Pre-LEV} = 0.0467$	$RMSE_{Pre-LEV} = 0.0467$
$RMSE_{Post-LEV} = 0.0257$	$RMSE_{Post-LEV} = 0.0544$	$RMSE_{Post-LEV} = 0.0544$

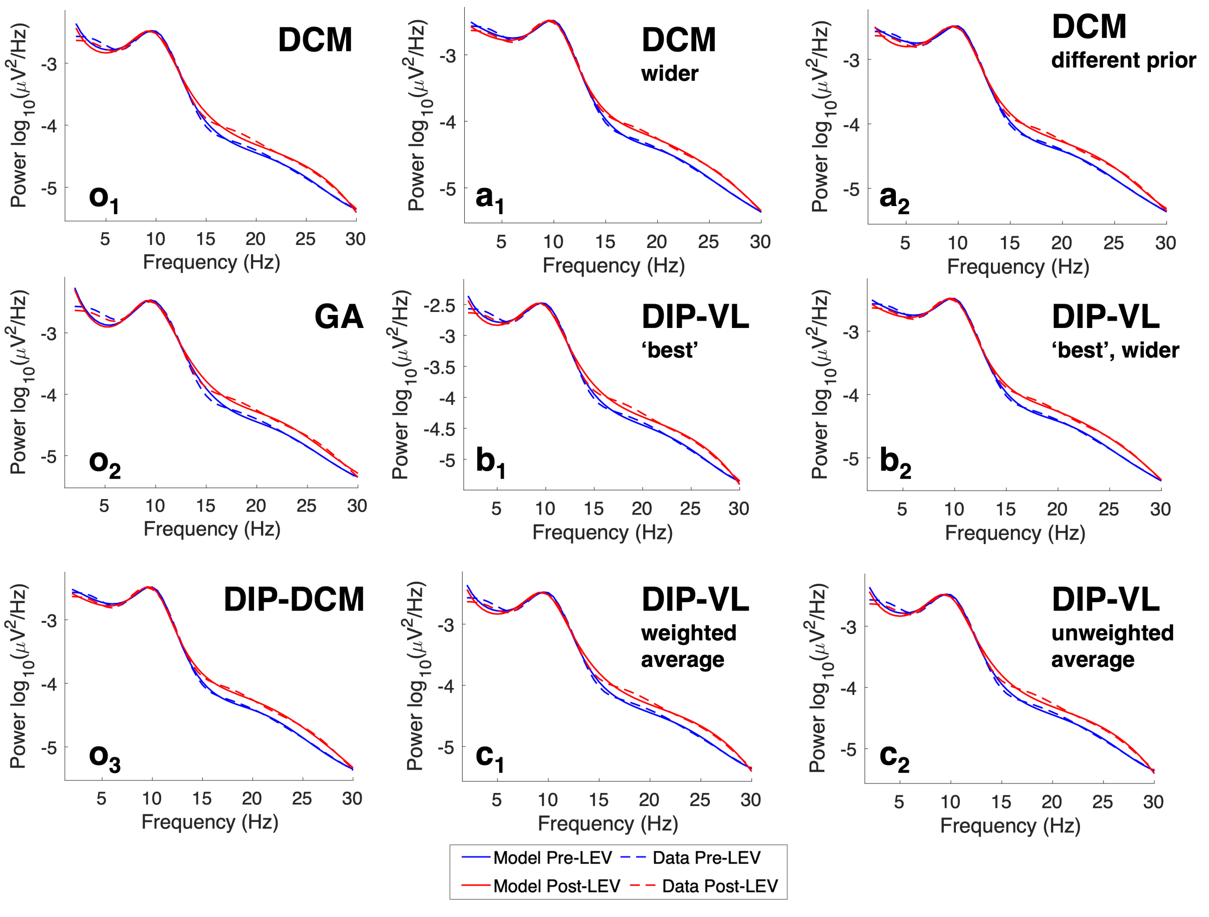

Figure S3: **Comparisons against alternative initialisation strategies: power spectra**. **A**: Data is shown as mean (for o_2, o_3, c_1, c_2). o_1, o_2 and o_3 correspond to DCM with "standard" priors, the genetic algorithm (GA) and the original DIP-DCM that uses GA estimates as priors for DCM. a_1 and a_2 correspond to analyses described in **3.1**, respectively, DCM with "standard" prior means and unit variances (a_1) and DCM with priors derived from the DIP-DCM bounds (a_2). b_1 and b_2 correspond to analyses described in **3.2**. c_1 and c_2 correspond to analyses described in **3.3**, respectively, the multi-start adaptation of DIP-DCM where each posterior forming the multi-modal distribution is weighted by model-evidence(c_1), or weighted by $1/500$ as default in DIP-DCM (c_2). *Abbreviations*: LEV, levetiracetam; DIP-DCM, dynamic causal modelling with dynamics-informed priors.

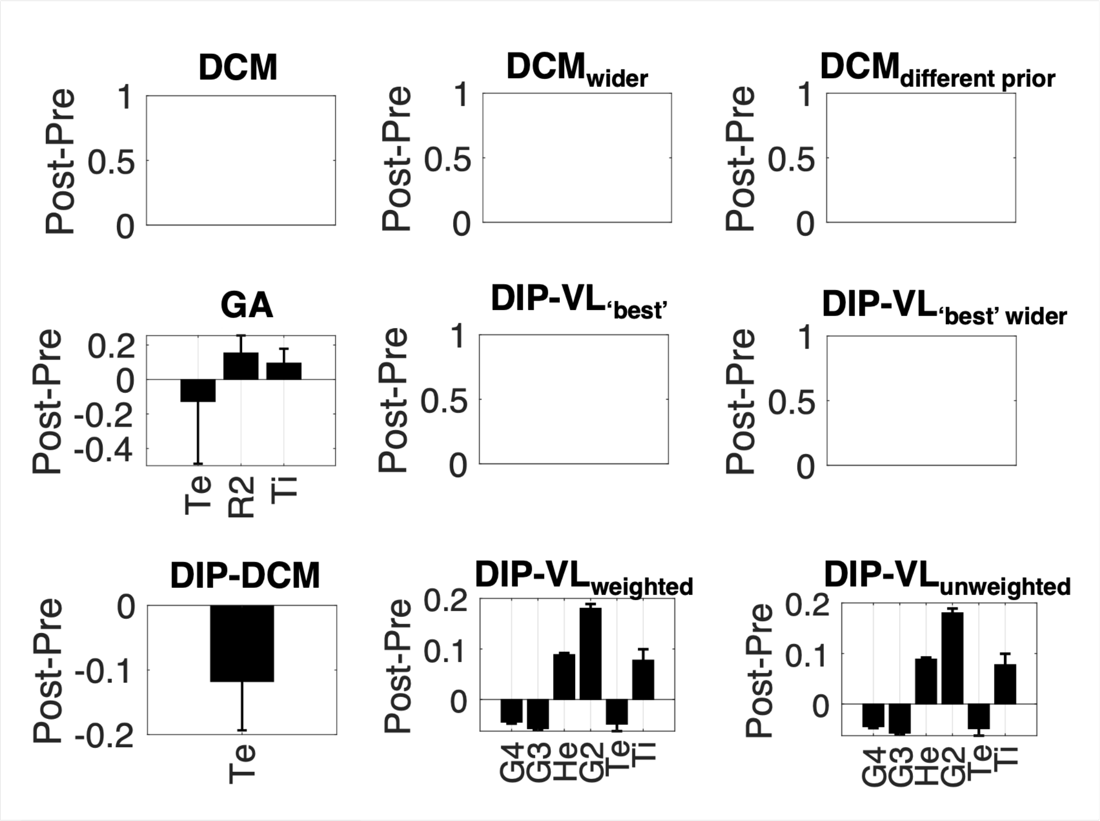

Figure S4: **Comparisons against alternative initialisation strategies: parameter inferences**. Mechanistic effects were obtained as the difference between the parameter distributions of Post-LEV and Pre-LEV conditions, and selected based on 95% Bayesian Credible Intervals and statistical power (Cohen's $d \geq 0.2$), as per **Methods 2.4**. The y-axis units of the parameter effects vary depending on the parameter. *Abbreviations*: LEV, levetiracetam; DIP-DCM, dynamic causal modelling with dynamics-informed priors.

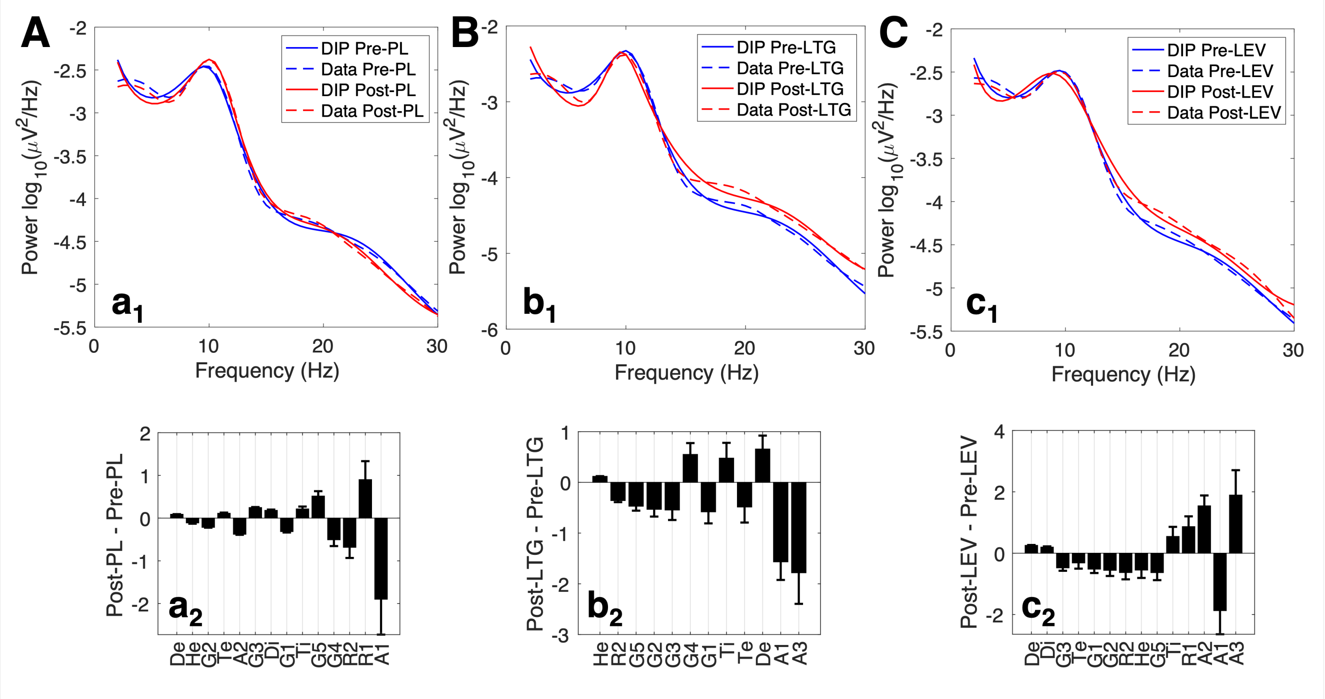

Figure S5: **DIP-DCM with "standard" variance.** DIP-DCM was run using the GA prior means and the variances from the "standard" SPM priors for the LFP model, rather than using unit variances. A: Power spectra of the model and data (a_1) and parameter effects (a_2) for the pre-placebo (PL) and post-placebo conditions. B: Power spectra of the model and data (b_1) and parameter effects (b_2) for the pre-lamotrigine (LTG) and post-lamotrigine conditions. C: Power spectra of the model and data (c_1) and parameter effects (c_2) for the pre-levetiracetam (LEV) and post-levetiracetam conditions. This figure can be compared to **Figure 3** to understand how the goodness of fit of the model and the parameter effects change when using the "standard" variances rather than unit variances. The y-axis units of the parameter effects vary depending on the parameter.

4 Supplementary 4

The performance the DIP-DCM parameter estimation approach was assessed via two complementary analyses (**Figure S6**). These analyses were performed using data from the placebo condition of **Study 1** (Biondi et al., 2022), serving as a baseline comparison.

First, the number of generations used in the global search – implemented in DIP-DCM as a genetic algorithm – was systematically reduced to determine the minimum required to produce stable model dynamics. Notably, 150 generations seemed to be necessary to initialise the DCM variational inference sufficiently close to the optimum and achieve optimal model fitness, as measured via root mean square error (RMSE). This configuration also maintained moderate computation times for each individual inversion (**Figure S6A**). Notably, DIP-DCM outperformed a genetic algorithm alone as well as a DCM initiated from randomly sampled priors using a Latin Hypercube (LH), highlighting the best trade-off between accurate estimation and computational efficiency.

Second, with the number of generations fixed at 150, the number of priors, and therefore the number of variational inversions, was systematically reduced from 500 to 50 (**Figure S6B**) and selected based on the RMSE fitness scores. Notably, when the number of priors was reduced below 400, there was a gradual decline in parameter inference accuracy, with spurious (confounding) effects beginning to emerge at 100 and 50 inversions. Mechanistic effects were calculated from the estimated parameter distributions as per **Methods 2.4**. Results suggest that a sufficiently large number of inversions is required to average out confounds and obtain statistically reliable estimates. However, it is also possible that improved methods for selecting informative priors could mitigate these effects.

5 Supplementary 5

Figure S7 is an additional example of a "ground truth" parameter recovery on synthetic data (**Results 3.1**). In this example, the "ground truth" is not only a single value, but regions of the parameter space.

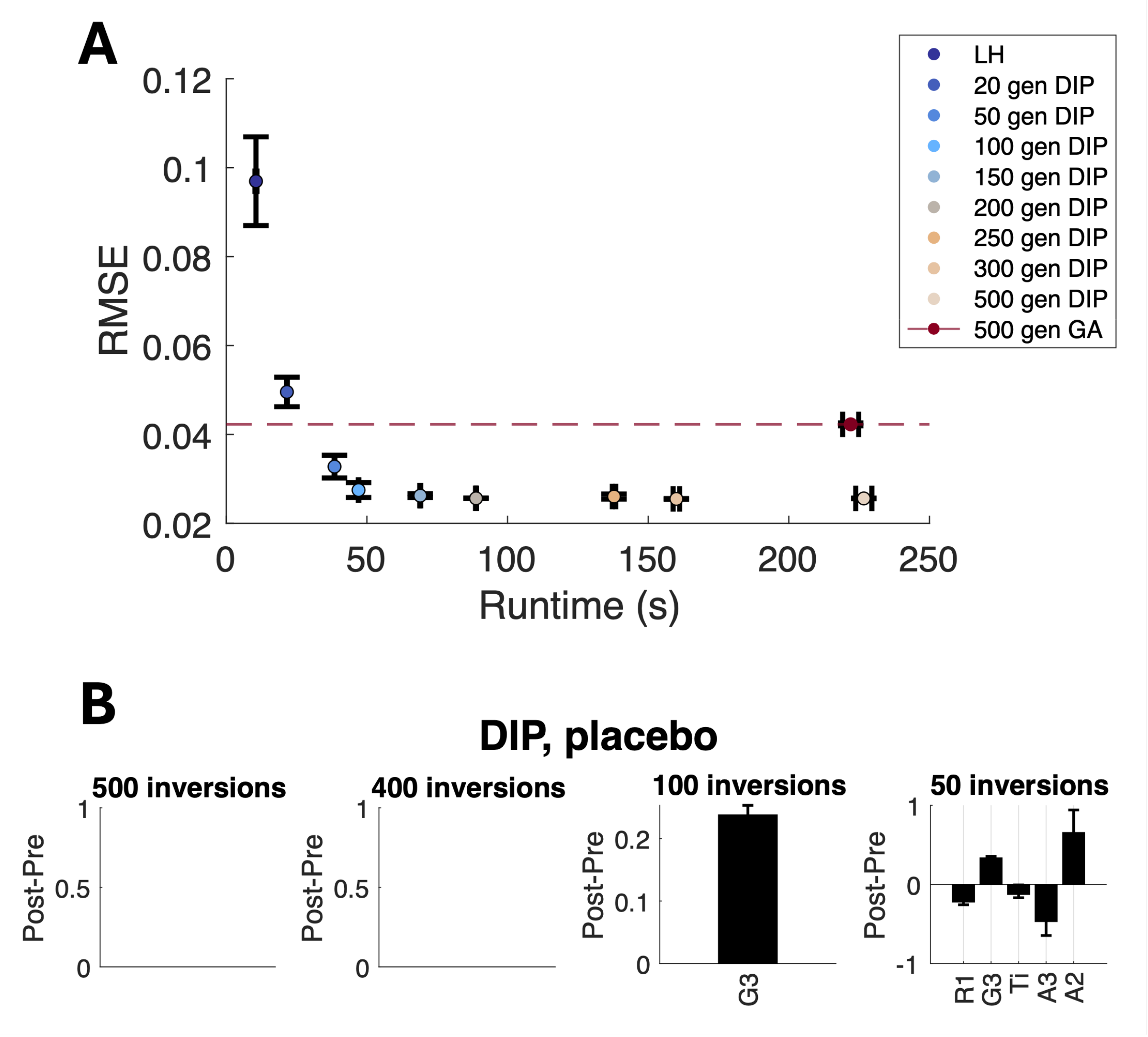

Figure S6: **Efficiency of the dynamics-informed approach**. **A**: Systematic reduction in the number of generations used by the genetic algorithm (GA). RMSE is the root mean square error between spectra of model and data for the pre-placebo experimental group, runtime is for each GA or DIP-DCM inversion (n=500). Data is shown as mean $\pm$ S.E.M. **B**: With the number of generations fixed at 150, the number of priors was systematically reduced from 500 to 50 in steps of 50. Placebo effects were calculated using the estimated parameter distributions as per **Methods 2.4** and shown as mean differences and 95% bayesian credible intervals. The y-axis units of the parameter effects vary depending on the parameter. *Abbreviations*: PL, placebo; gen, generations; DIP-DCM, dynamic causal modelling with dynamics-informed priors; LH, Latin hypercube.

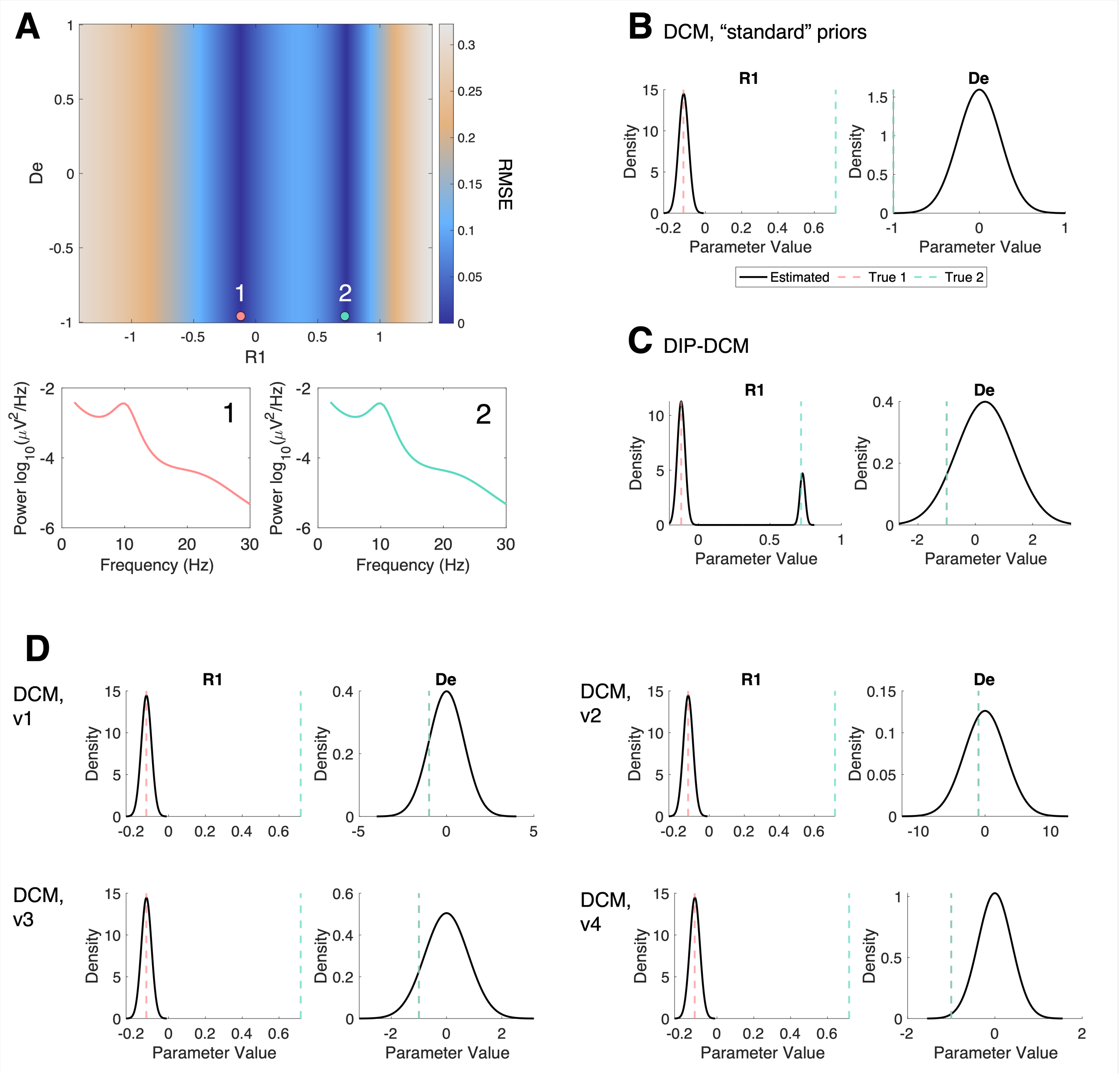

Figure S7: **Parameter recovery on synthetic data.** **A**: RMSE mapping illustrating two regions of the parameter space where the model has equal dynamics. Parameter bounds are those used in DIP-DCM. **B**: Parameter recovery using DCM with "standard" priors, DIP-DCM, and DCM variants (v1,"standard" mean and unit variance; v2,"standard" mean and wider variance; v3,"standard" mean and wider variance, which was proportional to the variance of the "standard" priors; v4, priors derived from the DIP-DCM bounds, where the prior mean is in the middle of the interval and the variance spans 99% of the interval). Model fitting was conducted on the synthetic data corresponding to the parameter set '1' in **A**. *Abbreviations*: DIP-DCM, dynamic causal modelling with dynamics-informed priors; DCM, dynamic causal modelling.

6 Supplementary 6

Correlations between parameters estimated with DCM or DIP-DCM are reported in **Figure S8**, for both the 'reduced' and 'full' models (**Supplementary 1.4**). DCM and DIP-DCM display distinct and weakly correlated posterior parameter structures, and none of the two methods seem to substantially reduce or inflate the parameter dependencies.

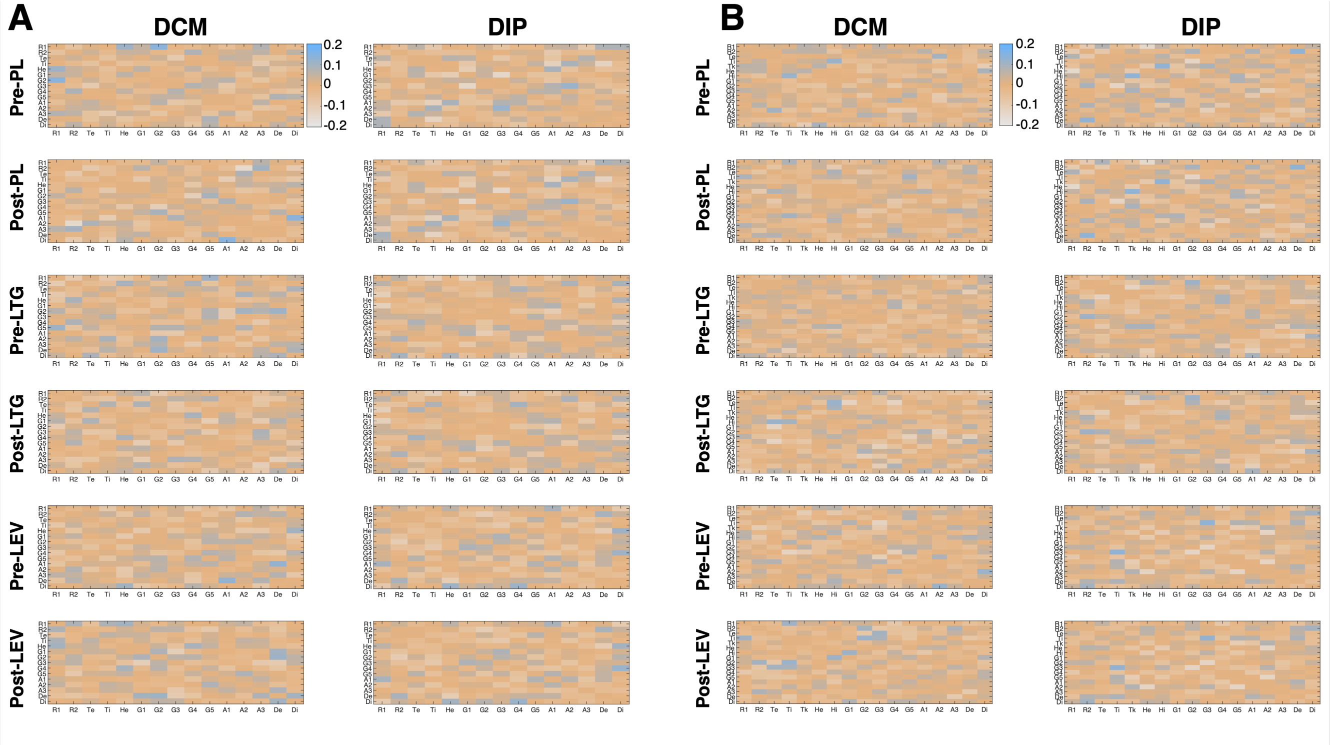

Figure S8: **Parameter correlations for the 'full' (A) and 'reduced' (B) models.** Correlations were calculated between the posterior parameters estimated with DCM and DIP-DCM and the colourmap represents Pearson's correlation coefficients and is consistent for all plots. *Abbreviations*: DCM, dynamic causal modelling; DIP-DCM, DCM with dynamics informed priors.

Bibliography of Supplementary Material

References

Biondi, A., Rocchi, L., Santoro, V., Rossini, P. G., Beatch, G. N., Richardson, M. P., & Premoli, I. (2022). Spontaneous and tms-related eeg changes as new biomarkers to measure anti-epileptic drug effects. *Scientific Reports*, *12*(1). https://doi.org/10.1038/s41598-022-05179-x

Deb, K. (2001). *Multi-objective optimization using evolutionary algorithms*. Wiley.

Dunstan, D., Richardson, M., Abela, E., Akman, O., & Goodfellow, M. (2023). Global nonlinear approach for mapping parameters of neural mass models. *PLoS Computational Biology*, *19*(3 March). https://doi.org/10.1371/journal.pcbi.1010985

Friston, K., Harrison, L., & Penny, W. (2003). Dynamic causal modelling. *NeuroImage*, *19*(4), 1273–1302. https://doi.org/10.1016/S1053-8119(03)00202-7

Friston, K., Kahan, J., Biswal, B., & Razi, A. (2015). A dcm for resting state fmri. *Neuroimage*, *94*, 396–407. https://doi.org/10.1016/j.neuroimage.2013.12.009

Friston, K., Parr, T., Zeidman, P., Razi, A., Flandin, G., Daunizeau, J., Hulme, O., Billig, A., Litvak, V., Moran, R., Price, C., & Lambert, C. (2019). Dynamic causal modelling of covid-19. *Wellcome Open Research*, *5*, 89. https://doi.org/10.12688/wellcomeopenres.15881.2

Moran, R., Kiebel, S., Stephan, K., Reilly, R., Daunizeau, J., & Friston, K. (2007). A neural mass model of spectral responses in electrophysiology. *NeuroImage*, *37*(3), 706–720. https://doi.org/10.1016/j.neuroimage.2007.05.032

Moran, R., Pinotsis, D., & Friston, K. (2013). Neural masses and fields in dynamic causal modelling. *Frontiers in Computational Neuroscience*. https://doi.org/10.3389/fncom.2013.00057

Moran, R., Stephan, K., Seidenbecher, T., Pape, H. C., Dolan, R., & Friston, K. (2009). Dynamic causal models of steady-state responses. *Neuroimage*, *44*, 796–811. https://doi.org/10.1016/j.neuroimage.2008.09.048

Novelli, L., Friston, K., & Razi, A. (2024). Spectral dynamic causal modeling: A didactic introduction and its relationship with functional connectivity. *Netw Neurosci*, *8*(1), 178–202. https://doi.org/10.1162/netn_a_00348

Oppenheim, A., Willsky, A., & Young, I. (1983). *Signals and systems*. Englewood Cliffs, N.J. Prentice-Hall.

Parr et al. T., G., Pezzulo, & Friston, K. (2022). *Active inference : The free energy principle in mind, brain, and behavior.* The MIT Press.

Razi, A., Kahan, J., Rees, G., & Friston, K. (2015). Construct validation of a dcm for resting state fmri. *Neuroimage*, *106*. https://doi.org/10.1016/j.neuroimage.2014.11.027

Shaw, A., Knight, L., Freeman, T., Williams, G., Moran, R., Friston, K., Walters, J., & Singh, K. (2020). Oscillatory, computational, and behavioral evidence for impaired gabaergic inhibition in schizophrenia. *Schizophrenia bulletin*, *46*(2), 345–353. https://doi.org/10.1093/schbul/sbz066

Zeidman, P., Friston, K., & Parr, T. (2023). A primer on variational laplace (vl). *NeuroImage*, *279*. https://doi.org/10.1016/j.neuroimage.2023.120310
